# Characterization of two novel bacteriophages (PSV6 and PSV3) as biocontrol agents against *Pseudomonas syringae*

**DOI:** 10.3389/fmicb.2025.1633072

**Published:** 2025-11-12

**Authors:** Shengting Zhang, Sha Zhao, Jinhua Li, He Zou, Yani Ju, Yangjian Xiang, Yunlin Wei

**Affiliations:** 1 School of Ethnic Medicine, Yunnan Minzu University, Kunming, China; 2 Faculty of Life Science and Technology, Kunming University of Science and Technology, Kunming, China

**Keywords:** bacteriophage therapy, *Pseudomonas syringae* pv. *syringae*, eco-friendly biocontrol, plant protection, pathogenic bacteria

## Abstract

*Pseudomonas syringae* is a prevalent plant-pathogenic bacterium. Conventional control strategies, such as fungicides, copper-based compounds, and antibiotics, often result in elevated environmental toxicity and promote the emergence of bacterial resistance. Consequently, bacteriophage-based biocontrol has garnered considerable attention as an eco-friendly alternative. In this study, two novel bacteriophages, PSV6 and PSV3, were isolated from wastewater using *Pseudomonas syringae* pv. *syringae* (Pss) as the host strain. Transmission electron microscopy and whole-genome phylogenetic analyses indicated that both phages belong to the class *Caudoviricetes*, with PSV6 classified within *Autotranscriptaviridae* and PSV3 within *Jondennisvirinae*. The one-step growth characteristics, as well as the thermal, pH, and solvent stabilities of PSV6 and PSV3, were further evaluated under different solvent conditions. Their host range, a critical factor for their environmental applications, was also assessed. Whole-genome sequencing revealed that the genome of PSV6 is 40,070 bp in length and contains 48 open reading frames (ORFs), whereas that of PSV3 is 229,871 bp long and comprises 376 ORFs, with no virulence factors or antibiotic resistance genes detected. *In vivo* assays in *Arabidopsis thaliana* demonstrated that increasing phage titers enhanced biocontrol efficacy against Pss. Notably, treatment with both phages was more effective than treatment with PSV6 alone. Moreover, treatment with PSV3 at a multiplicity of infection of 100 exhibited the strongest preventive and therapeutic effects against Pss. Overall, our results highlight the potential of PSV6 and PSV3 as sustainable alternatives to chemical pesticides in agriculture.

## 1 Introduction

Plant diseases caused by bacterial pathogens pose a major threat to global agriculture (Wang et al., 2022). Among them, *Pseudomonas syringae* pv. *syringae* (Pss) has been used as a model to improve our understanding of the molecular mechanism underlying bacterial pathogenicity and plant-microbial interactions (Xin et al., 2018) and is one of the most studied plant pathogens (Chen et al., 2022). It can infect more than 300 economically important crops, including legumes, crucifers, and members of the Rosaceae family. Moreover, *P. syringae* causes numerous plant diseases, such as halo blight in beans, bacterial canker in peach trees, and bacterial speck in tomatoes (Rabiey et al., 2020; Xie et al., 2020), leading to substantial economic losses worldwide. Traditional methods for controlling *P. syringae* include the use of fungicides, copper compounds, antibiotics, and other control methods. However, these methods not only fail to adequately control Pss but also lead to high environmental toxicity, promoting bacterial resistance (Pereira et al., 2021). Therefore, there is an urgent need for sustainable and environmentally friendly alternatives for bacterial infection management in plants.

Bacteriophages (phages), which are viruses that specifically infect and lyse bacteria, have gained considerable attention as potential biocontrol agents against plant bacterial diseases (Haq et al., 2024). Compared to traditional chemotherapy, phage therapy is more targeted, reduces the risk of disrupting normal microbiota during treatment, and is adaptable. Notably, phages can evolve alongside bacterial pathogens. This dynamic interaction allows them to maintain their effectiveness against bacteria over the long term (Koskella and Brockhurst, 2014). Over the past decade, phage-based biocontrol has been increasingly recognized as a promising solution for controlling plant diseases caused by bacterial pathogens, particularly *Pseudomonas* (De Smet et al., 2017), *Xanthomonas* (Liu et al., 2022a), and *Erwinia* species (Gayder et al., 2020). Phage treatment can significantly reduce bacterial populations in plant tissues, alleviate disease symptoms, and promote plant health (Fiorillo et al., 2023).

Numerous phages targeting *P. syringae* have been isolated from environmental sources such as soil, water, and plant surfaces. These include phages specific to *P. syringae* pv. *tomato* (Skliros et al., 2023), *P. syringae* pv. *phaseolicola* (Martino et al., 2021), and *P. syringae* pv. *actinidiae* (Psa) (Ni et al., 2021). These phages exhibit strong potential for treating plant bacterial diseases. For example, phages ph002F and ph004F, isolated from coffee plant leaves, are promising candidates for combating bacterial halo blight (Silva et al., 2023). Akbaba and Ozaktan, 2021 demonstrated, using *in vivo* and *in vitro* experiments, that treatment with these phage therapies can reduce the severity of cherry diseases and exhibits effective biocontrol activity against *P. syringae*. These findings provide a foundation for phage-based management of *P. syringae* bacterial diseases. However, phage activity can be significantly influenced by environmental factors, limiting their practical application in agricultural settings (Pinheiro et al., 2020). Moreover, only a few phages targeting Pss have been isolated and reported (Rabiey et al., 2020; Ivanović et al., 2017 and Song et al., 2021). Esearch on treatment conditions for Pss-induced bacterial infections, as well as evaluations of their practical application value, remains lacking. Therefore, isolating and characterizing novel phages that are well-adapted to natural environments is crucial for improving the stability and effectiveness of phage therapy. The identification of such phages would expand the range of phage-based treatment options. Moreover, a comprehensive study of their genetic and biochemical properties will facilitate the development of more effective and safer phage cocktail formulations, enabling efficient treatment of bacterial infections.

Therefore, the present study aimed to isolate and characterize two novel bacteriophages, PSV6 and PSV3, that infect Pss, elucidate their genomic structure, and evaluate their efficacy as biocontrol agents. Our findings contribute to the advancement of phage-based strategies for managing bacterial diseases in crops and provide valuable insights into the practical application of phage therapy.

## 2 Materials and methods

### 2.1 Strains and growth conditions

The strain used in the present study, Pss (strain number CGMCC 1.3070, GenBank entry number: AF094749), was purchased from the China General Microbiological Culture Collection Center (CGMCC). The strain was cultured in Luria–Bertani (LB) broth at 30° C with shaking at 180 rpm/min. It was preserved in 50% glycerol at −80° C for long-term storage.

### 2.2 Phage isolation, purification, and titer determination

Using Pss as the host bacteria, bacteriophages were isolated from sewage using a double-layer agar plate method, as previously described (Kyrkou et al., 2020). Briefly, sewage collected around hospitals in Kunming, Yunnan, was centrifuged at 15,000 × *g* for 15 min, and the resulting supernatant was filtered through a 0.22-μm microporous filter. A total of 1 mL of the filtrate was added to 5 mL of a log-growing host Pss culture, and the mixture was incubated overnight at 30 °C with shaking at 180 rpm and 4 °C. After centrifugation at 15,000 × *g* for 15 min, the supernatant was filtered again through a 0.22-μm microporous filter, and a phage stock was obtained. This phage stock solution was serially diluted with LB liquid medium, and 100 μ*L* of different dilutions of this bacteriophage solution was mixed with 300 μ*L* of Pss host cell culture. This mixture was incubated at 20–25 °C for 15 min, mixed with 4.5 mL LB semi-solid medium, and poured onto the surface of pre-prepared LB solid media. The phages were cultured at 30 °C overnight and monitored for plaque formation. Clear single plaques were selected and transferred into a host bacterial suspension [optical density at 600 nm (OD_600_) = 0.5–0.8] in the logarithmic growth stage for phage amplification. The mixture was incubated overnight at 30 °C with shaking at 180 rpm/min. Subsequently, the culture was centrifuged at 4 °C and 15,000 × *g* for 15 min, and the supernatant was filtered through a 0.22-μm microporous filter. Phage plaques were then assessed using the double-layer agar plate method. This purification step was repeated multiple times until only a single plaque morphology with consistent size appeared on the plates. The obtained filtrate was considered a pure bacteriophage solution, and its titer was subsequently determined using the double-layer agar plate method. The purified bacteriophage was stored in 50% glycerol at −80 °C and was regularly revived to maintain viability.

### 2.3 Determination of the optimal Multiplicity of Infection (MOI) of the phages

An optimal MOI represents the ratio of phages to bacteria at the time of infection. To determine the MOI, host bacteria were cultured to the logarithmic growth phase until they reached a colony number of 3 × 10^7^ colony-forming units [CFU]/mL, as previously described (Liu et al., 2024). Phage stock with a known titer and host cells at the exponential stage were mixed with a gradient of MOI ranging from 0.001–100 and cultured in a shaker at 180 rpm/min at 30 °C for 12 h. The phage titer was then determined using the double-layer agar plate method after centrifugation (15,000 × *g* for 15 min at 4° C), followed by filtration through a 0.22-μm membrane. The experiment was repeated three times, and the average value was obtained, with the highest value representing the optimal MOI.

### 2.4 Transmission Electron Microscopy (TEM)

#### 2.4.1 Phage concentration

To concentrate the phages, a 1% inoculum of the Pss seed culture (OD_600_ = 1) was transferred into 500 mL of LB liquid medium and cultured at 30° C with shaking at 180 rpm/min until the culture reached the logarithmic growth phase. The phages were then added at an optimal MOI (of 1), and the culture was further incubated until the medium became clear, indicating bacterial lysis. Subsequently, phage lysates were collected.

#### 2.4.2 Phage purification

The phage-containing culture was cooled to 26° C, followed by the addition of DNase I and RNase A to a final concentration of 1 μg/mL. The mixture was incubated at room temperature for 30 min to degrade residual host nucleic acids. Solid NaCl was then added to a final concentration of 1 mol/L, mixed until fully dissolved, and the mixture was incubated in an ice bath for 1 h. The mixture was then centrifuged at 15,000 × *g* for 10 min at 4° C to remove cellular debris. The resulting supernatant was collected, and its volume was measured. Polyethylene glycol (PEG) 6,000 was added to a final concentration of 10% (w/v). The mixture was stirred to dissolve PEG and placed in an ice-water bath overnight for phage particle precipitation. After centrifugation at 15,000 × *g* for 15 min at 4° C, the phage pellet was collected, and the supernatant was discarded. The phage pellet was resuspended in SM buffer (100 mM NaCl, 10 mM MgSO_4_, 50 mM Tris-HCl at pH 7.5, and 0.01% gelatin) and incubated at room temperature for 1 h. An equal volume of chloroform was then added to extract PEG and cell debris from the phage suspension. The resulting mixture was gently shaken for 30 s and centrifuged at 3,000 × *g* for 10 min at 4° C. The hydrophilic phase containing the phage particles was recovered, and the extraction process was repeated two to three times.

To purify phage particles, cesium chloride (CsCl) was added to the phage suspension to a final concentration of 0.6–0.75 g/mL, and the mixture was stirred until CsCl was completely dissolved. The solution was transferred to ultracentrifuge tubes and overlaid with an SM solution containing 0.75 g/mL CsCl. The tubes were centrifuged at 1,50,000 × *g* for 8 h at 4° C. After centrifugation, the phage bands were carefully extracted using a 1 mL syringe. Each band was collected in 0.5–1 mL fractions and stored at 4° C until use.

#### 2.4.3 Phage morphology observation using TEM

Phage morphology was observed using a negative staining method with 2% phosphotungstic acid. Phage particles were adsorbed onto copper grids for 5–10 min, air-dried, and stained with 2% phosphotungstic acid for 2 min as previously described (Abdelghafar et al., 2023). The samples were observed using low-voltage TEM.

### 2.5 One-step growth curve generation

The parameters of the one-step growth curve were determined based on a previously described method (Liu et al., 2021). Briefly, phages were added to a log-phase culture of Pss (3 × 10^7^ CFU/mL) to achieve an MOI of 1, and the mixture was incubated for 15 min at room temperature. After centrifugation at 15,000 × *g* for 5 min at 4° C, the supernatant was discarded. The resulting phage-infected bacteria pellet was washed three times with LB medium at 30° C. The final pellet was resuspended in 30 mL of LB medium and incubated at 30° C with shaking at 180 rpm/min. Samples (600 μ*L*) were collected every 15 min, centrifuged, and filtered. The phage titer was then determined using the double-layer agar plate method. Control groups without phages or bacteria were included for comparison. The experiment was repeated three times, and the results were plotted to determine the phage latent period, lysis period, and steady phase.

### 2.6 pH and thermal stability of the phages

To assess the effects of temperature and pH on the survival of the PSV6 and PSV3 phages, 10 μ*L* of PSV6 and PSV3 lysates (1–3 × 10^9^ plaque-forming units [PFU]/mL) were added to 990 μ*L* of various aseptic buffers, including citrate, phosphate, Tris-HCl, glycine, carbonate, and sodium hydrogen phosphate buffers, with pH levels ranging from 3–12 and incubated at 30 °C for 2 h. After dilution, the phage titers were determined using the double-layer agar plate method. For the thermal stability experiments, 4 mL of phage stock solution was incubated at varying temperatures (4, 30, 40, 50, 60, and 70 °C) for 180 min. Samples were collected every 30 min, and phage titers were determined. All experiments were performed in triplicate.

### 2.7 Effect of organic solvents on phage survival

The effect of organic solvents on phage survival was determined using a previously reported method (Pallavi et al., 2021) with some modifications. Briefly, 900 μ*L* of PSV6 or PSV3 (1–3 × 10^9^ PFU/mL) was mixed with ether, chloroform, acetone, or ethanol. The mixture was incubated for 30 min, followed by centrifugation at 15,000 × *g* for 10 min. The supernatant was then diluted, and the phage titers were measured using the double-layer agar method, with the LB medium used as a control. The experiment was repeated three times.

### 2.8 Phage host range

The host range of the Pss phages PSV6 and PSV3 was assessed using the spot assay method. Bacterial strains included *P. syringae* ATCC 19304, *P. aeruginosa* ATCC 10145, *Bacillus cereus* ATCC 14579 and GDMCC 1.3589, *Serratia marcescens* ATCC 13880, and *Escherichia coli* ATCC 11303, which were standard strains purchased from the Strain Preservation Center. Additionally, *Serratia marcescens* KMR-3 was an isolated strain. For each assay, 200 μ*L* of bacterial cells were mixed with 4.5 mL of a semi-solid medium and transferred onto the surface of a double-layer agar plate. A total of 10 μ*L* of the phage suspension (10^9^ PFU/mL) was spotted onto the double-layer agar surface of different bacterial lawns. The plates were incubated at 28° C overnight, and plaque formation within the spotted areas was observed. The experiment was repeated three times.

### 2.9 Genome sequencing and analysis of phages PSV6 and PSV3

Phage genomic DNA was extracted using an OMEGA-Viral DNA kit 50 (Guangzhou Feiyang Biological Engineering Co., Ltd, China) following the manufacturer's instructions. The extracted phage genomes were treated with DNase I and RNase A to determine the type of nucleic acid present. To verify whether the genomes of PSV6 and PSV3 are circular or linear, three pairs of reverse primers for PSV6 and two pairs of reverse primers for PSV3 were designed, along with one pair of forward primers as a positive control. The sequences of primers are listed in Table 1. PCR amplification was performed using the phage DNA as the template. The presence of bands indicated circular DNA, whereas their absence suggested linear DNA.

**Table 1:** Design of primers for validating genome type.

**Primers names**	**Sequence(from 5^′^to 3^′^)**	**bp**
PSV6-1	F: CATGCAGGCTCAATTCAGGTC R: CGCGTGATACGCGAATATGAC	934
PSV6-2	F: CATCACCTCCTTATAGGAGCACTG R: GTGAACTGTACGCACCTTGAGAC	1,241
PSV6-3	F: GATCTAAACACGTTAGCGACACG R: CGTTGTTGGCGATCATTCTACAG	1,625
PSV3-1	F: GTTATGCGAAGTCTTCGAAGTGTC R: GGCCGAAGTAATTAAGCAAGGTC	1,017
PSV3-2	F: GCAGCGAATAACGTTCTTCAAACG R: GTCGTCGCATATATTGCGCAAG	1,597
PSV3-3 (Positive control)	F: GTATATTGCTTACGTATCCAGCGG R: CAATACACGACGCTAGACCAAG	1,110

The extracted PSV6 and PSV3 genomic DNA were subjected to next-generation sequencing by Shenggong Bioengineering (Shanghai) Co., Ltd. using an Illumina Novaseq6000 sequencing platform (Illumina Inc., San Diego, CA, USA). Raw data were evaluated using FastQC, and the sequences were trimmed using Trimmomatic to obtain high-quality and reliable data. After quality control, genome assembly was performed using SPAdes software (https://github.com/ablab/spades). Subsequent bioinformatic analyses were conducted on the assembled sequences to obtain and characterize the complete the whole genome of PSV6 and PSV3. Whole-genome was mapped using GCView (https://cgview.ca/) (Grin and Linke, 2011), and a whole-genome phylogenetic tree was constructed using the VICTOR database (https:genomes//ggdc.dsmz.de/victor.php#). The phylogenetic trees of the terminase and major capsid proteins of PSV6 and PSV3 were generated using the neighbor-joining method in MEGA-X software (Kumar et al., 2018). The VFDB (http://www.mgc.ac.cn/VFs/) virulence factor database and antibiotic resistance gene database (https://card.mcmaster.ca/) were screened for genes associated with virulence factors and antibiotic resistance genes (Chen et al., 2016).

### 2.10 *In vitro* lysis of pss by phages

To assess the effects of PSV6 and PSV3 on Pss and generate lysis curve experiments, a 1–2% inoculum of Pss was cultured in 50 mL LB liquid medium at 30 °C with shaking at 180 rpm/min until it reached the logarithmic growth phase. PSV6 and PSV3 phages were then added to the culture at MOIs of 0.001, 0.01, 0.1, 1, 10, and 100, and incubation continued under the same conditions. A total of 200 μ*L* of culture was sampled hourly, and the OD_600_ was measured using a microplate reader. The control group included a host bacterial suspension without phages or a phage suspension without host bacteria. Three replicates were performed for each group, and the experiment was repeated three times.

### 2.11 Pathogenic model establishment

To determine whether Pss was pathogenic to *Arabidopsis thaliana*, a pathogenicity model was established.

#### 2.11.1 Arabidopsis seed planting

*Arabidopsis* seeds were purchased from Yunnan Chi'en Technology Co., Ltd. The seeds were surface-sterilized by soaking in 10% NaClO for 5 min, followed by 3–5 washes with sterile water, immersion in 75% ethanol for 1 min, and five washes with sterile water. Sterilized seeds were sown on Murashige and Skoog (MS) solid medium plates, with 30–40 seeds per plate. The plates were maintained at 4° C for 3–5 d to induce seed vernalization. They were then transferred to a plant growth chamber at 25° C under a 16-h/8-h light/dark cycle for 1 week. Finally, the seedlings were transferred into 350 mL culture bottles containing MS medium and grown in a sterile culture room for 3 weeks.

#### 2.11.2 Bacterial suspension preparation

Pss strains stored at −80° C were streaked onto LB solid plates and incubated at 30° C until single colonies appeared. A single colony was then transferred into 5 mL of LB liquid medium and cultured at 30° C with shaking at 180 rpm/min until OD_600_ reached 1.0. The seed culture was transferred as a 1–2% inoculum in 100 mL LB medium, and the bacteria were cultured at 30° C with shaking at 180 rpm/min until the logarithmic growth phase was reached. The bacterial culture was centrifuged at 4° C and 8,000 × *g* for 10 min, and the supernatant was discarded. The pellet was resuspended in 10 mmol/L MgCl_2_ buffer and centrifuged 3–4 times. The final pellet was resuspended in 10 mmol/L MgCl_2_ buffer containing 0.02% Silwet L-77, and the OD_600_ was adjusted to 0.2 (approximately 10^7^ CFU/mL). The resulting suspension was used immediately.

#### 2.11.3 Arabidopsis seedling immersion inoculation

Using a previously described protocol (Ishiga et al., 2017) with some modifications, the prepared bacterial suspension (40 mL) was inoculated onto 3 week old *Arabidopsis* seedlings (adjusted from the original 2 week old *Arabidopsis* seedlings in the corresponding reference) via immersion at room temperature for 1–2 min (decreased from the reported 2–3 min). A control group was included with seedlings treated with 10 mmol/L MgCl_2_ buffer containing 0.02% Silwet L-77. After immersion inoculation, the *Arabidopsis* tissue-cultured seedlings were transferred to the growth chamber to observe and record leaf symptoms. The experiment was repeated three times.

### 2.12 *In vivo* phage biocontrol efficacy

The main challenge in the biological control of plant bacterial diseases lies in demonstrating the efficacy of bacteriophages for plant control or treatment. In this study, *A. thaliana* was used as a plant model to evaluate the biocontrol efficacy of PSV6 and PSV3. A positive control was prepared using 15 mL of 10 mmol/L MgCl_2_ buffer, and a negative control was established using 15 mL of the Pss suspension. Based on the *in vitro* experimental results, the three highest MOI values (1, 10, and 100) were selected for the experimental groups. Mixtures of Pss bacterial suspension and either single phages or a phage mixture (prepared by mixing PSV3 and PSV6 at equal titers in a 1:1 ratio) were prepared at a final volume of 15 mL, based on the different MOI values, and incubated for 15 min. The mixtures from each group were then poured onto 3 week old *Arabidopsis* seedlings for 1 min. The mixtures were then removed, and the seedlings were transferred to a sterile plant growth chamber for cultivation, with six replicates per group. Disease symptoms on the leaves were observed and recorded every 24 h. For each treatment, three randomly selected leaf discs (with diameters of 0.6 cm) were placed in 2 mL centrifuge tubes containing 1 mL of 10 mmol/L MgCl_2_ buffer. The leaf discs were ground in a sterile mortar, and 300 μL of the homogenate was serially diluted in 10 mmol/L MgCl_2_ buffer to concentrations of 10^−1^, 10^−2^, 10^−3^, 10^−4^, and 10^−5^. Subsequently, 100 μL of each dilution was spread on LB agar plates and incubated at 30° C for 24 h to count the colony-forming units (CFUs). This experiment was repeated three times.

### 2.13 Statistical analyses

Statistical analyses were performed using GraphPad Prism software (GraphPad Inc., La Jolla, CA, USA). One-way analysis of variance (ANOVA) was used to evaluate significant differences in phage titer under different MOIs and organic solvents and determine significant differences in biocontrol efficacy under different experimental conditions. *P* < 0.05 was considered statistically significant, and three independent replicates were performed for each condition.

## 3 Results

### 3.1 Morphology of phages PSV6 and PSV3

**Figure 1:**
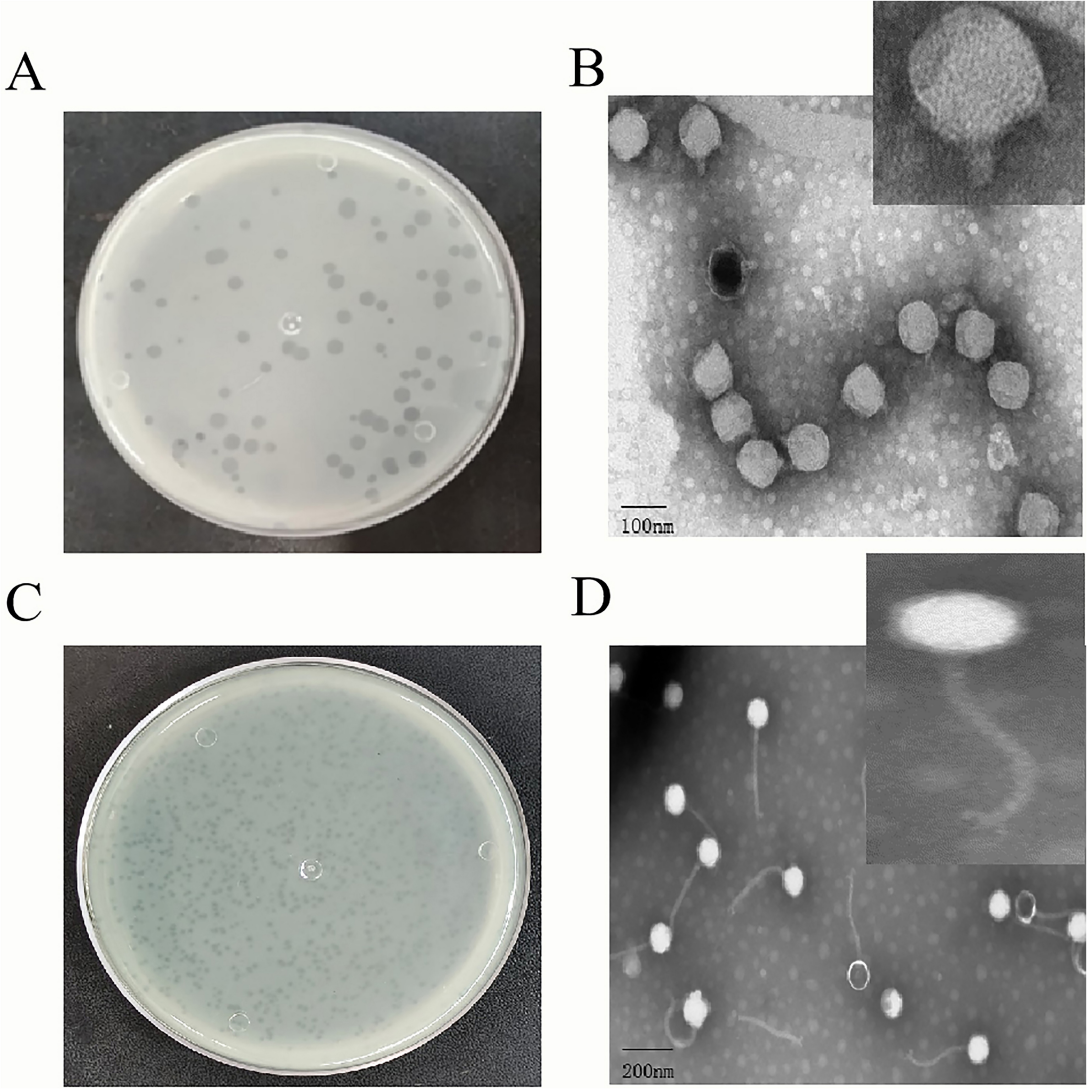
Morphological characteristics of *Pseudomonas syringae* pv. *syringae* phages PSV6 and PSV3. **(A)** Phage plaque morphology of PSV6 on an LB double-layer agar plate. **(B)** Transmission electron microscopy (TEM) image of PSV6. **(C)** Phage plaque morphology of PSV3 on an LB double-layer agar plate; **(D)** TEM image of PSV3.

To identify phages that can control Pss infections, we used Pss as a host for two strongly lytic phages isolated from wastewater, PSV6 and PSV3. Using the double-layered plate, we observed clear plaques on the lawn of Pss. The plaques formed by PSV3 were significantly smaller than those of PSV6, with diameters of 0.5–1 mm and 1–3 mm, respectively. Both plaques were transparent without any halos (Figures 1A, C). TEM revealed that the morphologies of the two phages differed. Specifically, the head of the PSV6 phage was icosahedral with a diameter of 54 ± 1 nm, and it had a short, non-contractile tail with a diameter of 22 ± 1 nm (Figure 1B). In contrast, the head of PSV3 was polyhedral with a diameter of 45 ± 1 nm, and it had a long, non-contractile tail with a diameter of 186 ± 1 nm, where the tail fibers were clearly visible (Figure 1D).

### 3.2 Biological characteristics of PSV6 and PSV3

**Figure 2:**
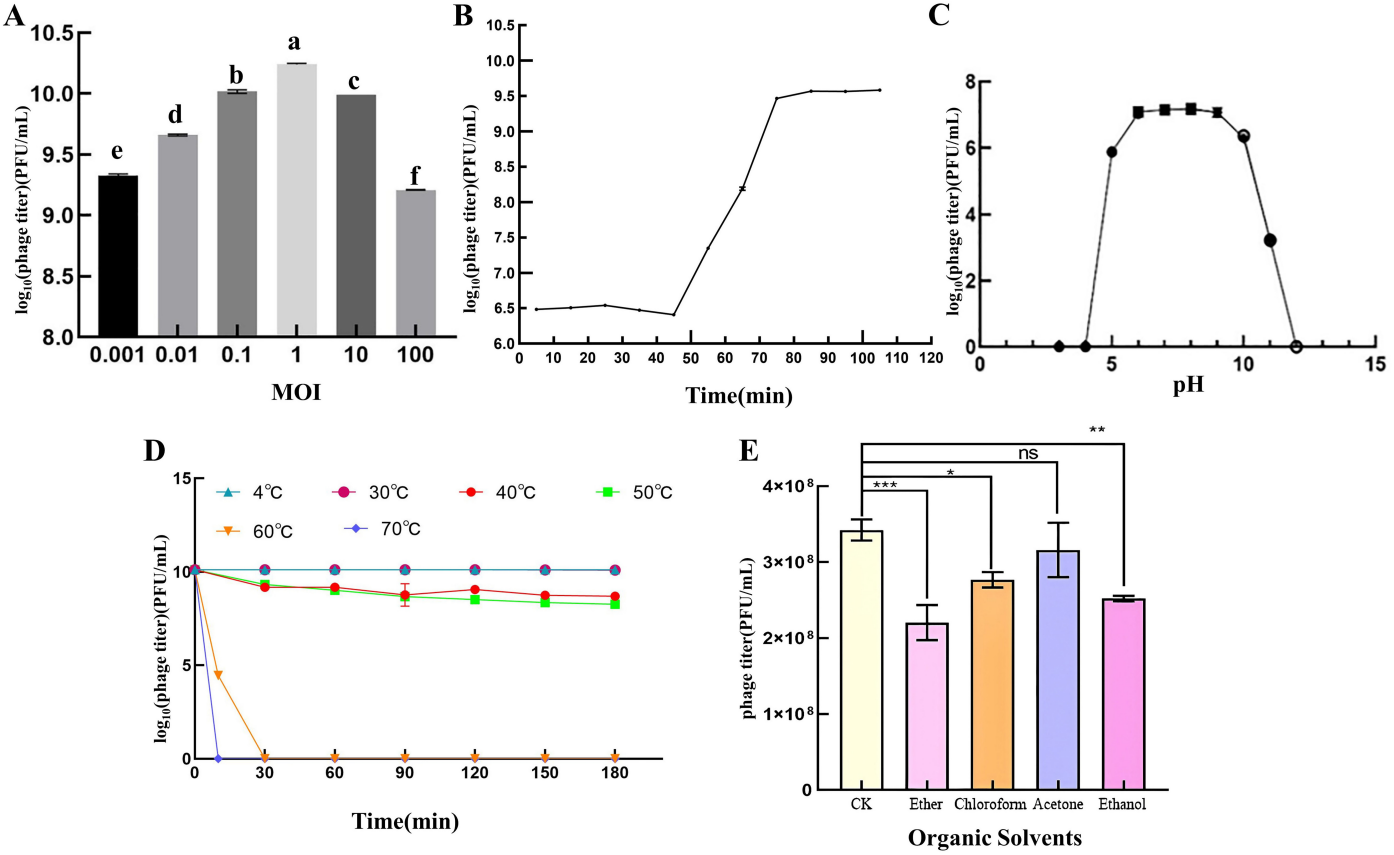
Biological features of the PSV6. **(A)** Titers at different multiplicities of infection. One-way ANOVA was performed, and different letters above the error bars indicate significant differences. **(B)** One-step growth curve. **(C)** pH stability. **(D)** Temperature stability. **(E)** Effect of organic solvents on phage survival. Values are means from three independent experiment. Error bars represent standard deviation. One-way analysis of variance (ANOVA) was used to assess statistical significance, ns: no significant difference, *P < 0.05, **P < 0.01, ***P < 0.001. Each assay was performed in triplicate. Values represent averages, and error bars represent standard deviation.

**Figure 3:**
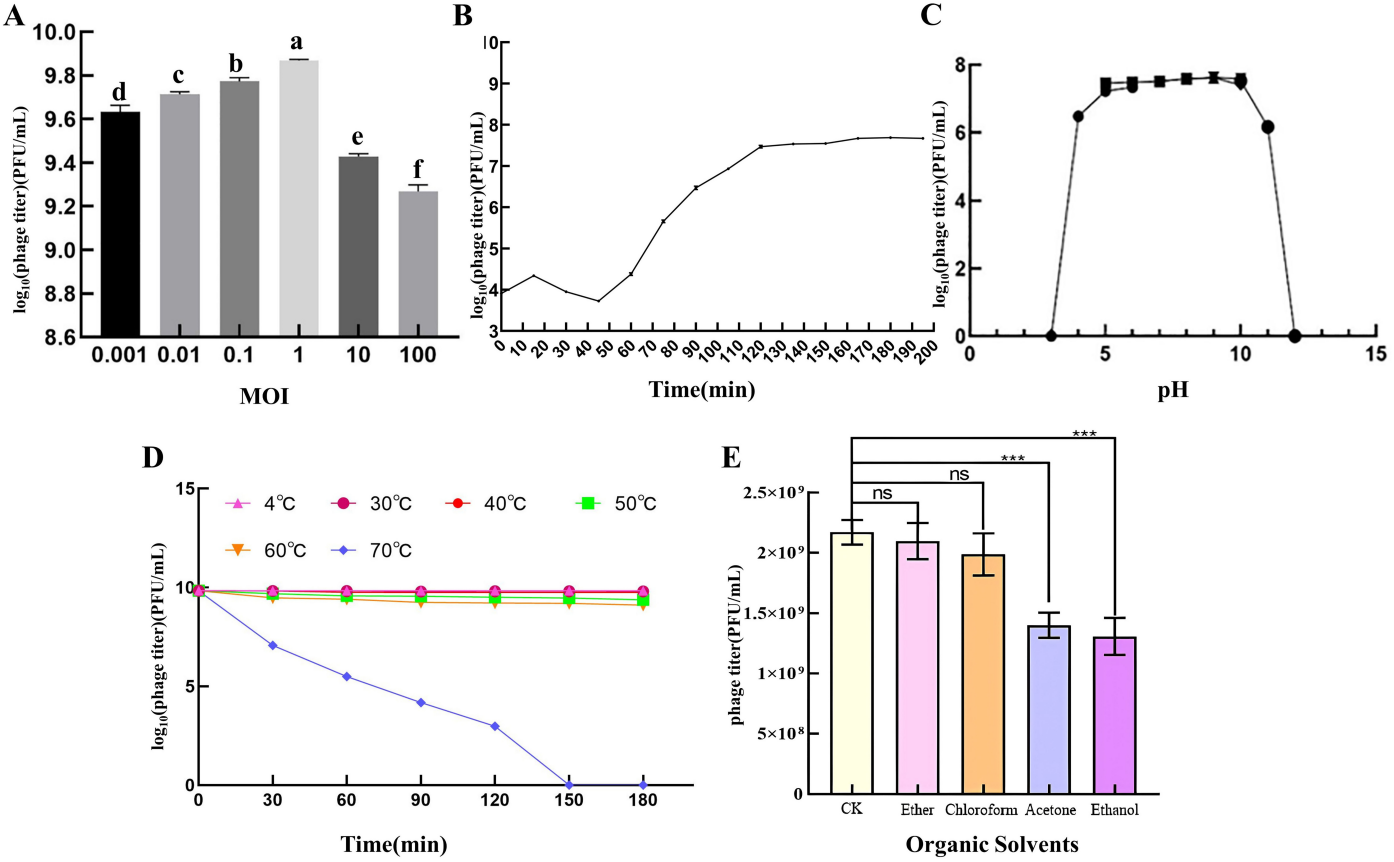
Biological features of PSV3. **(A)** Titers at different multiplicities of infection. One-way ANOVA was performed, and different letters above the error bars indicate significant differences. **(B)** One-step growth curve. **(C)** pH stability. **(D)** Temperature stability. **(E)** Effect of organic solvents on phage survival. Values are means from three independent experiments. Statistical significance was assessed using an unpaired Student's *t*-test, ns: no significant difference. *P < 0.05, **P < 0.01, ***P < 0.001. Each assay was performed in triplicate. Values represent averages, and error bars represent standard deviation.

When the MOI of PSV6 and PSV3 was 1, the number of progeny phages released by Pss infected by phages was the highest (1.74 × 10^10^ PFU/mL and 7.40 × 10^9^ PFU/mL, respectively), indicating that the optimal infection complex numbers of both PSV6 and PSV3 were 1 (Figures 2A, 3A). The incubation period, ascent period, and burst size are key parameters used to characterize the life cycle of phages (Sillankorva et al., 2008). Our one-step growth curve demonstrated that these three values for PSV6 were 40 min, 40 min, and 135 PFU/cell (Figure 2B), whereas they were 45 min, 75 min, and 16 PFU/cell for PSV3, respectively (Figure 3B). The pH stability tests showed that PSV6 remained stable within the pH range of 6–9 (Figure 2C), while PSV3 was stable between pH 5 and 10 (Figure 3C); however, both phages were inactivated at extreme pH values. PSV3 exhibited greater pH stability than PSV6. In terms of temperature stability, PSV6 maintained its titer between 4–30° C (Figure 2D), while PSV3 was stable over a broader range of 4–60° C (Figure 3D). Both phages showed a gradual decline in titer with increasing temperature, with PSV3 demonstrating better thermal stability than PSV6. Moreover, the sensitivities of PSV6 and PSV3 to organic solvents slightly differed. Specifically, PSV6 was resistant to acetone but was sensitive to chloroform, ether, and ethanol (Figure 2E). PSV3 was resistant to chloroform and ether but sensitive to acetone and ethanol (Figure 3E). Among the seven different bacterial strains tested, only *P. syringae* ATCC 19304 was susceptible to PSV6 and PSV3, whereas the others were not infected by these phages (Table 2).

**Table 2:** Host ranges of phage PSV6 and PSV3.

**Strain**	**Lytic activity**	
	**PSV6**	**PSV3**
*Pseudomonas syringae pv. syringae* (primary host)	**++**	**++**
*Pseudomonas syringae* ATCC 19304	**+**	**+**
*Pseudomonas aeruginosa* ATCC 10145	-	-
*Bacillus cereus* ATCC 14579	-	-
*Bacillus cereus* GDMCC1.2779	-	-
*Escherichia coli* ATCC 11303	-	-
*Serratia marcescens* ATCC 13880	-	-
*Serratia marcescens* KMR-3	-	-

“**++**”*: phage was efficient to infect host; “***+**”*: phage was little efficient to infect host; “–”: phage was not to infect host*.

### 3.3 Genomic characterization of PSV6 and PSV3

**Figure 4:**
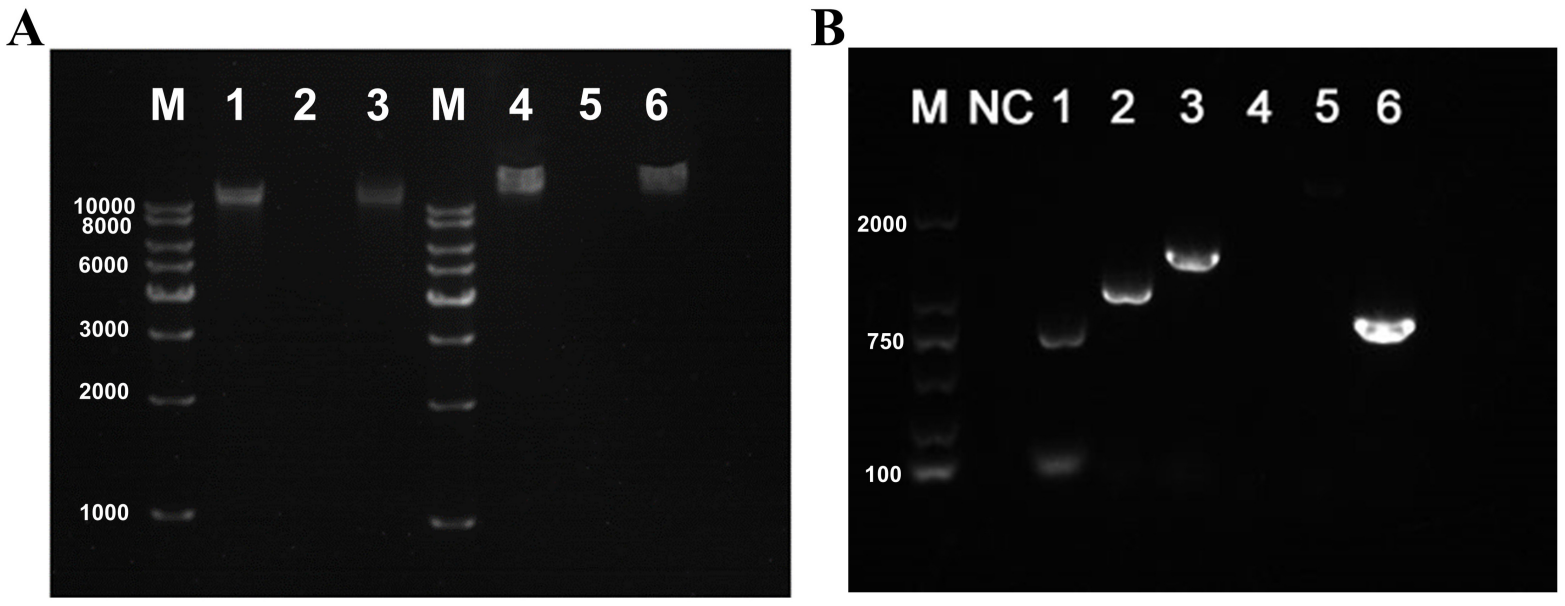
Verification of nucleic acid types and genomic morphology of PSV6 and PSV3. **(A)** Genome digestion patterns of phage PSV6 and PSV3. M: 1 Kb DNA Marker; 1: Untreated PSV6 genome; 2: PSV6 genome digested with DNase I; 3: PSV6 genome digested with RNase A; 4: Untreated PSV3 genome; 5: PSV3 genome digested with DNase I; 6: PSV3 genome digested with RNase A. **(B)** Validation of genomic DNA morphology for phages PSV6 and PSV3. M: 2000 bp DNA Marker; NC: Negative control (ddH_2_O replacing PSV6 template DNA); 1–3: Three different reverse primers for PSV6; 4–5: Two different reverse primers for PSV3; Lane 6: Positive control for PSV3 (forward primer pair).

The genomic nucleic acids of both phages could only be digested by DNase I but not by RNase A (Figure 4A). Moreover, the PSV6 reverse primer successfully amplified a band using genomic DNA as the template, whereas the PSV3 reverse primer failed to produce any amplification (Figure 4B). Therefore, the nucleic acid type of PSV6 is circular DNA, while that of PSV3 is linear DNA.

**Figure 5:**
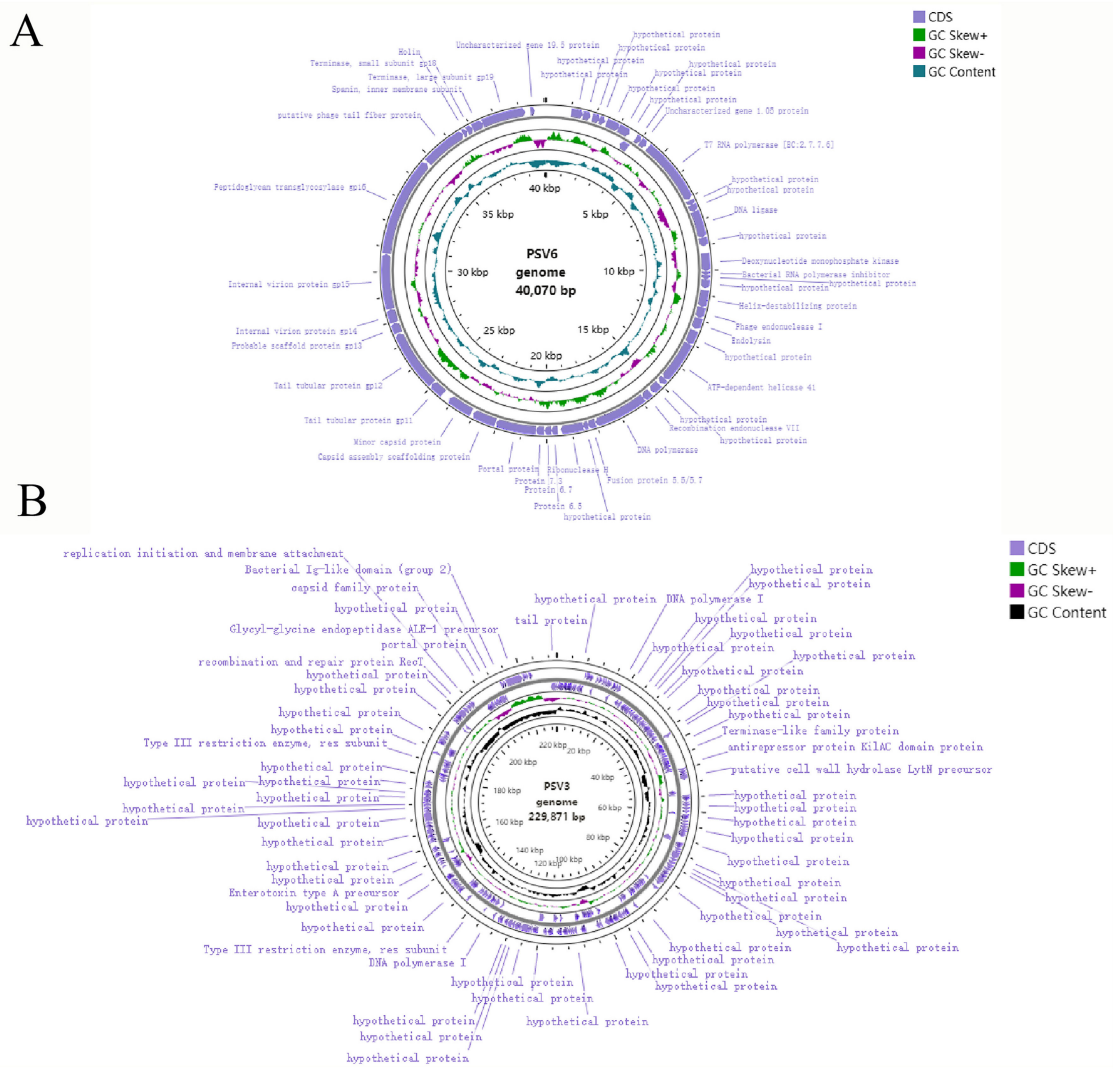
Whole-genome map. **(A)** PSV6. **(B)** PSV3.

**Figure 6:**
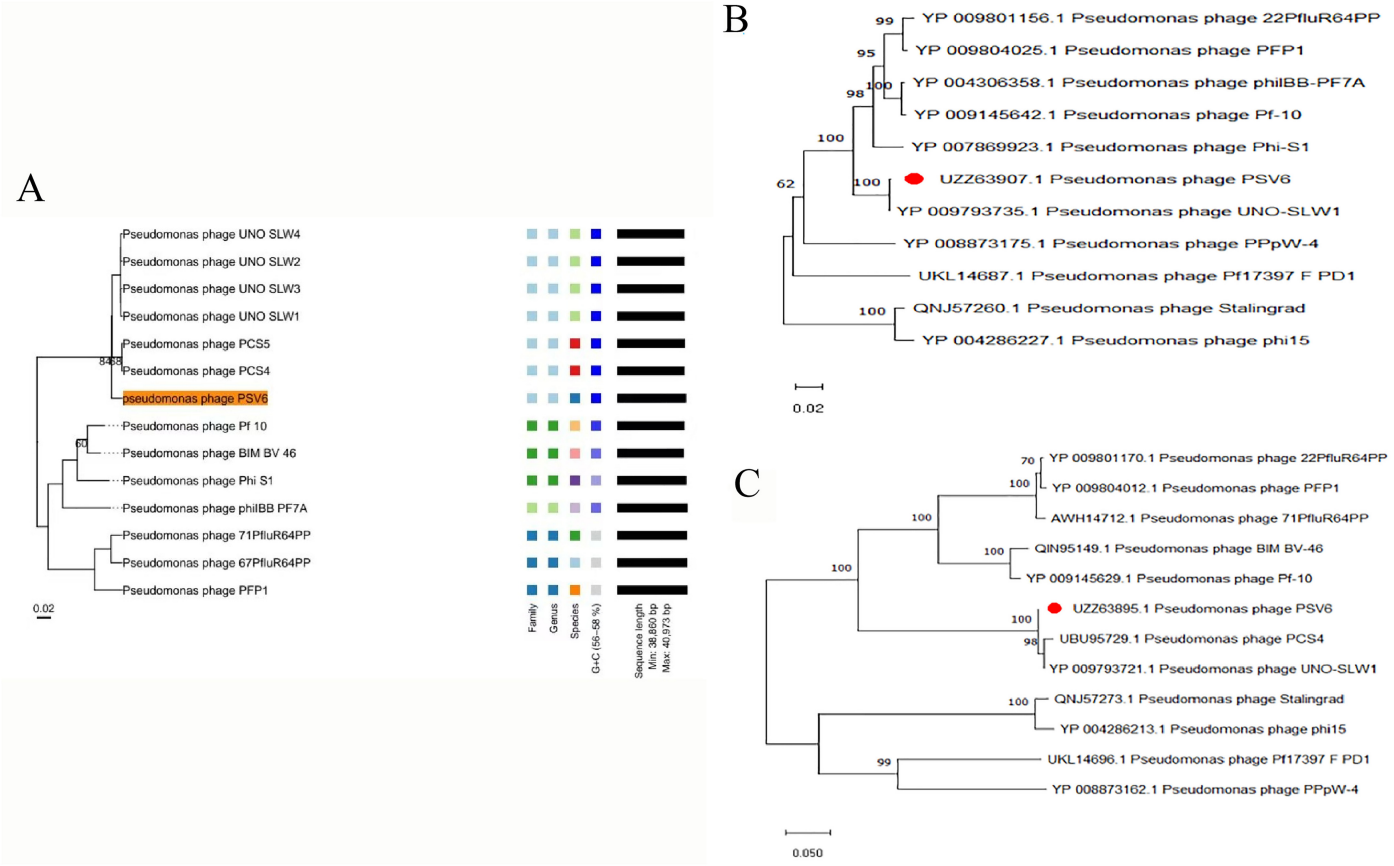
Phylogenetic trees of PSV6 using **(A)** whole-genome sequences (the right side of the figure indicates the classification levels: family, genus, species, and GC content similarity); **(B)** terminase large subunits, and **(C)** major capsid protein, with the neighbor-joining method.

**Figure 7:**
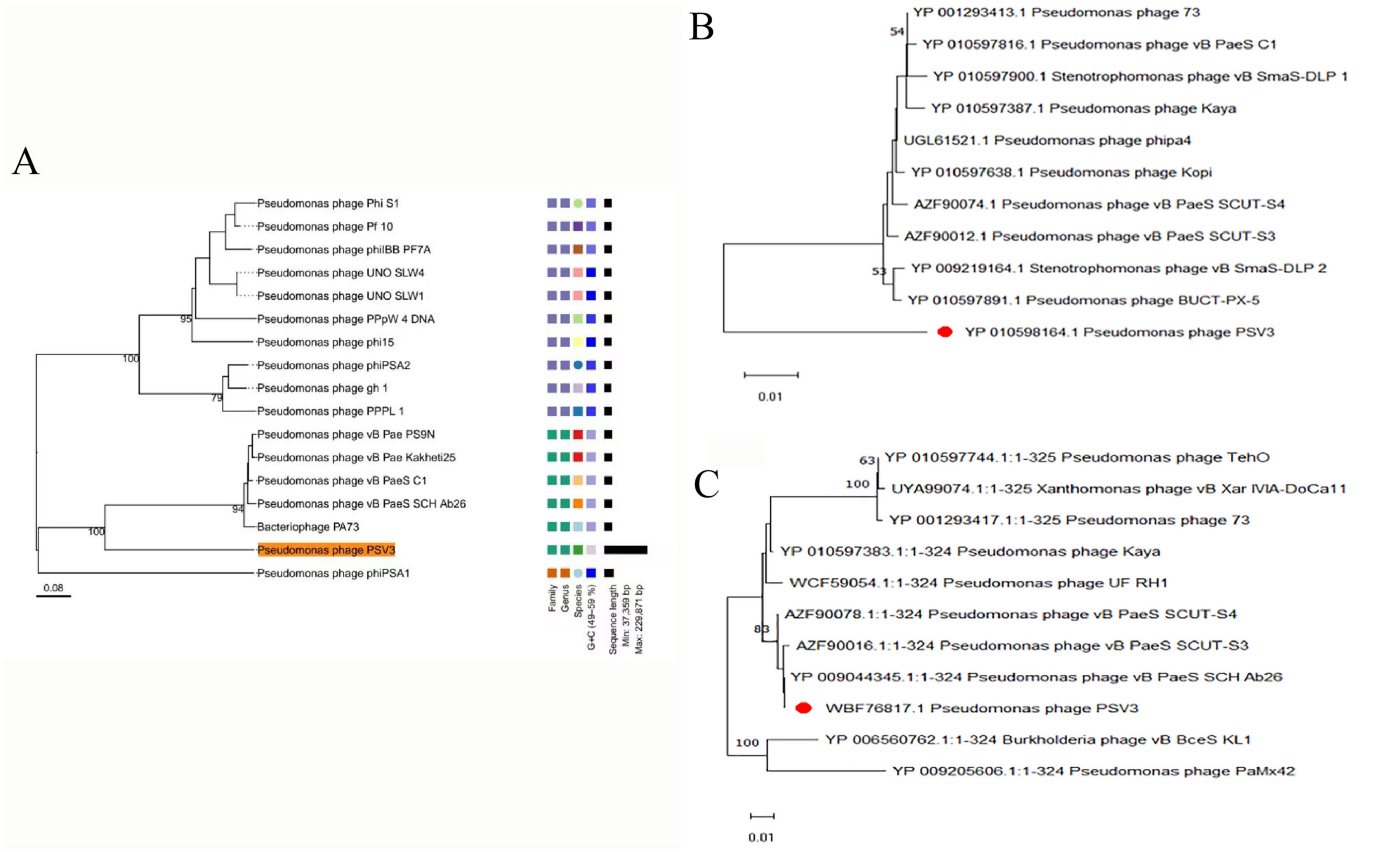
Phylogenetic tree of PSV3 using **(A)** Whole-genome sequences (the right side of the figure indicates the classification levels: family, genus, species, and GC content similarity); **(B)** terminase large subunit, and **(C)** Major capsid protein, with the neighbor-joining method.

To better understand phages and their applicability for biological control, PSV6 and PSV3 were subjected to whole-genome sequencing analysis. Our TEM and whole-genome phylogenetic tree analyses indicated that phages PSV6 and PSV3 both belong to the class *Caudoviricetes*, with PSV6 classified under *Autotranscriptaviridae* and PSV3 under *Jondennisvirinae*. The PSV6 genome (GenBank entry number: OP485124) has a length of 40,070 bps and 57.96% G + C content (Figure 5A). It also contains 48 open reading frames (ORFs), among which 31 are known functional coding sequences, and 17 are hypothetical proteins. The PSV3 genome (GenBank entry number: OP712460) has a total length of 2,29,871 bps and 49.08% G+C content (Figure 5B). It also contains 376 ORFs, among which 34 are known functional coding sequences and 341 are hypothetical proteins. Blast comparison analysis revealed that PSV6 is closely related to phages UNO-SLW1 to UNO-SLW4, PCS5, and PCS4, belonging to the same genus as them but not the same species (Figure 6A). PSV3 is located on the same branch as phages PSN9, Kakheyi25, PaeS C1, PaeS SCH Ab26, and PA73, sharing a common evolutionary origin and belonging to the same genus but not the same species (Figure 7A). The large subunit of the phage terminal enzyme and the major capsid protein are important for phage evolution. Therefore, we constructed evolutionary trees of the PSV3 and PSV6 terminal enzyme large subunits and major capsid proteins. The results indicated that the terminal large subunit of PSV6 exhibits high homology with UNO-SLW1, PPpW-4, 22PfluR64PP, PFP1, phiIBB-PF7A, Pf-10, and Phi-S1 in the existing NCBI database (Figure 6B). The main capsid proteins demonstrated high homology with PCS4, UNO-SLW1, 22PfluR64PP, PFP1, 71PfluR64PP, BIM BV-46, and Pf-10 in the database (Figure 6C). The terminal large subunit of PSV3 is closely related to the bacteriophages vB_SmaS-DLP_1 and BUCT-PX-5 (Figure 7B). The main capsids are closely related to the bacteriophages vB_PaeS_SCH_Ab26 and vB_PaeS_SCUT-S3 (Figure 7C) and belong to the same evolutionary clade. Moreover, our analysis of virulence factor and antibiotic resistance databases revealed that the PSV6 and PSV3 genomes did not encode virulence factors or antibiotic resistance-related genes; therefore, they could be used for subsequent biological control research.

### 3.4 Phage cleavage ability *in vitro*

**Figure 8:**
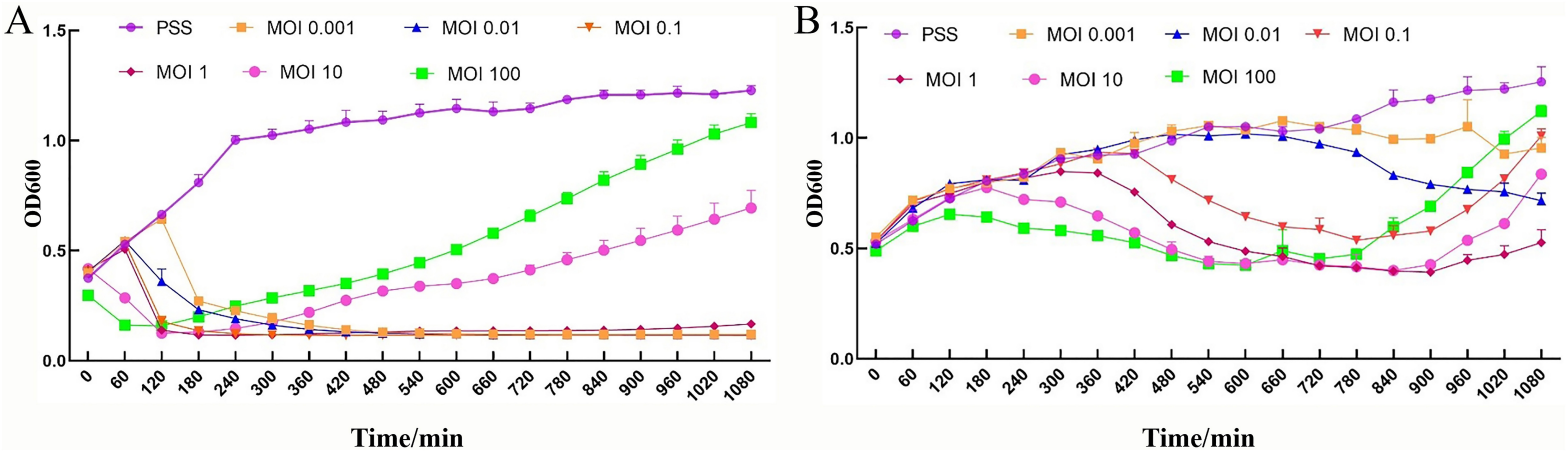
Phage cleavage curve. Growth inhibitory curve of *Pseudomonas syringae* pathovar syringae mixed with PSV6 and PSV3 at various MOIs. **(A)** PSV6: 0 (Pss control group, purple), 0.001 (yellow), 0.01 (blue), 0.1 (orange), 1 (red), 10 (pink), and 100 (green). **(B)** PSV3: 0 (Pss control group, purple), 0.001 (yellow), 0.01 (blue), 0.1 (orange), 1 (red), 10 (pink), and 100 (green). Each assay was performed in triplicate. Values represent averages, and error bars represent standard deviation.

To investigate the growth inhibitory effect of PSV6 and PSV3 on the host bacteria Pss, we determined the absorbance values of PSV6 and PSV3 at OD_600_ under different infection conditions. The two tested bacteriophages effectively reduced the OD value of the bacteria. At high titers (MOIs of 10 and 100), PSV6 and PSV3 decreased the OD value within a short period. However, the time required for bacterial OD was also short, with durations of 3 and 10 h for PSV6 and PSV3, respectively. At low titers, the OD value decreased slowly. At MOI 1 and MOI 0.1, PSV6 started to decrease the bacterial OD value from 2 h, whereas at an MOI 0.001, the bacterial OD value started decreasing at 3 h. However, at MOI 1, PSV6 notably resulted in a more rapid and pronounced decrease in bacterial OD (Figure 8A). At an MOI of 1, PSV3 notably decreased the bacterial OD value after 5 h, whereas at MOIs of 0.001 and 0.1, the bacterial OD value decreased after 11 h and 9 h, respectively (Figure 8B). Additionally, the OD value increased at low phage concentrations.

### 3.5 *In vivo* efficacy of phage treatment

**Figure 9:**
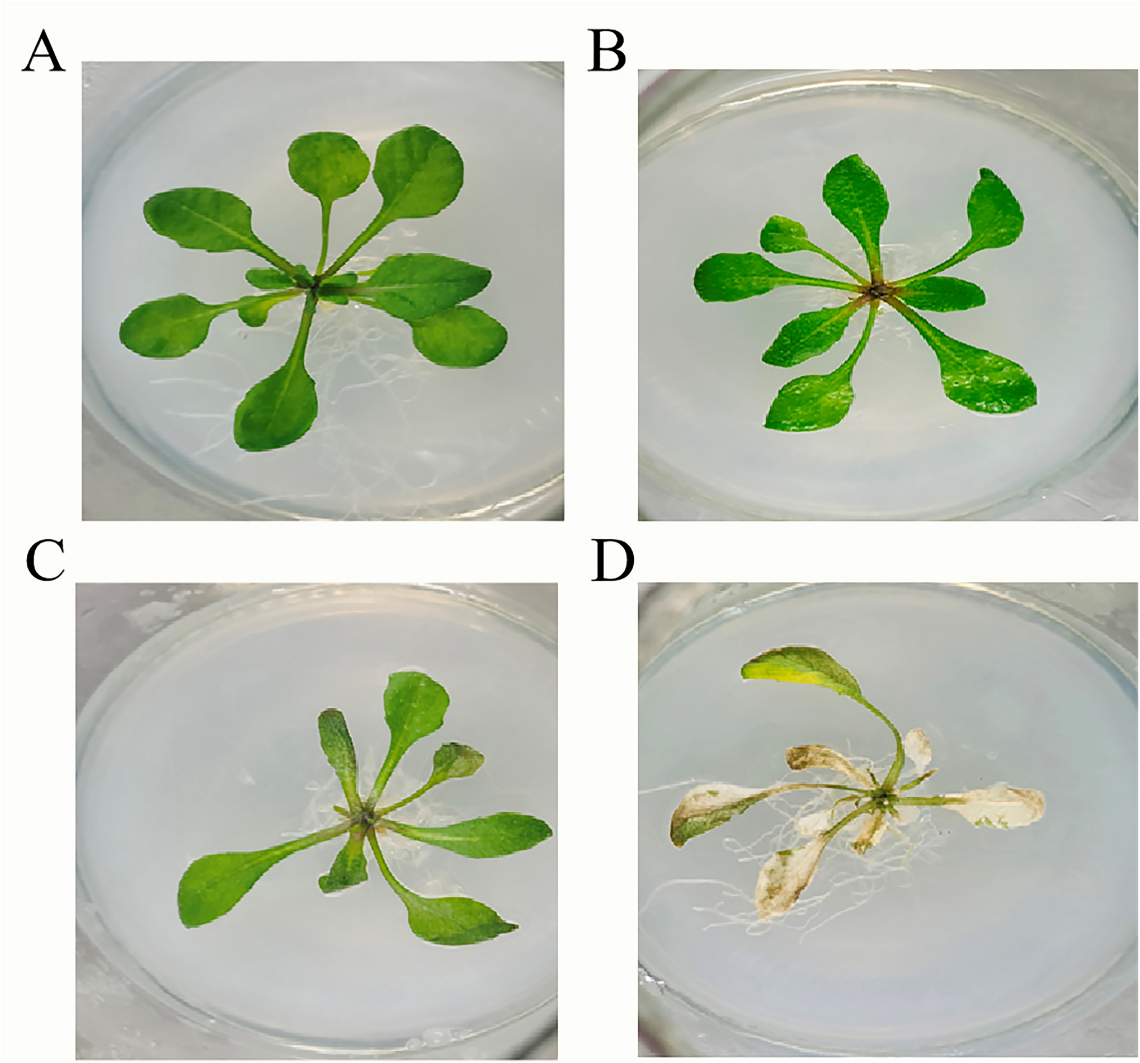
Pathogenicity of *Pseudomonas syringae* pv. *syringae* in *Arabidopsis*. **(A)** Control. **(B)** Flood inoculation at 0 h. **(C)** Flood inoculation at 24 h. **(D)** Flood inoculation at 96 h.

**Figure 10:**
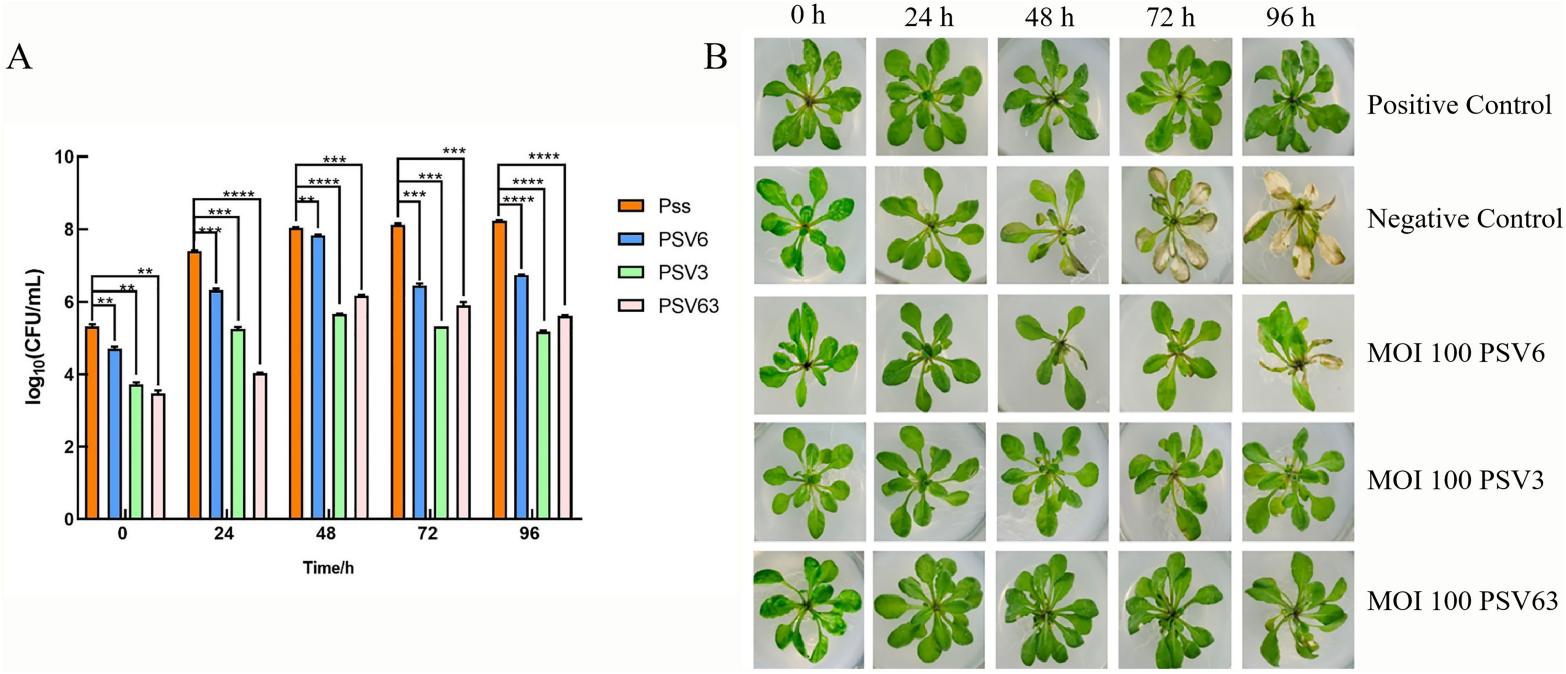
Control effect of phage treatment on *Pseudomonas syringae* pv. *syringae* (Pss) at an MOI 100 for 15 min. **(A)** Different phage treatments inhibit Pss at different times: Pss (orange), PSV6 (blue), PSV3 (green), and PSV6 and PSV3 co-treatment (pink). Values are means from three independent experiment. One-way analysis of variance (ANOVA) was used to assess statistical significance, ns: no significant difference, *P < 0.05, **P < 0.01, ***P < 0.001, ****P < 0.0001. **(B)** Bacteriophage control effect on Pss. The positive control was treated with 10 mmol/L MgCl_2_ buffer, and the negative control was treated with exponential phase Pss.

To evaluate the *in vivo* efficacy of PSV6 and PSV3, we developed a plant infection model using Pss and *A. thaliana* (Figure 9). Analysis of the effect of phage treatments at different MOIs for 15 min revealed that treatment with PSV6 at an MOI of 1 significantly decreased the number of Pss cells at 0–96 h of treatment, whereas PSV3 only significantly decreased the number of Pss cells at 24 h. Notably, longer sampling times exerted no significant effect on the number of Pss cells. The combination treatment with PSV6 and PSV3 resulted in a higher bacterial abundance at 24 h than that in the Pss group (negative control), and the number of bacteria was also significantly reduced at the remaining time points (Supplementary Figure 1A). We also observed leaf symptoms. Specifically, disease severity was lower in the PSV6 treatment group than in the PSV3 alone and combined phage treatment groups. The PSV3 alone and combined phage treatment groups exhibited symptom levels comparable to those in the Pss group from 48 to 72 h (Supplementary Figure 1C). At an MOI of 10, the three treatment groups exhibited significantly reduced numbers of Pss cells from 0 to 96 h; this effect was more significant than that observed at MOI 1. Moreover, PSV3 reduced the number of Pss more than PSV6 or the combination treatment (Supplementary Figure 1B). After 15 min of treatment at an MOI of 10, fewer *A. thaliana* leaves turned yellow at 96 h in the PSV3 treatment group than in the Pss group. From 48 to 96 h of treatment, the disease symptoms were more severe in the PSV6 and mixed treatment groups than in the PSV3 treatment group, but less severe than those in the Pss group (Supplementary Figure 1C). After treatment with phages at an MOI of 100 for 15 min, plants in the PSV6, PSV3, and mixed treatment groups demonstrated significantly reduced numbers of Pss cells compared to those treated at an MOI of 1 and 10. This finding indicates that an increase in phage concentration resulted in a more pronounced inhibitory effect on Pss *in vivo*. Moreover, PSV3 reduced Pss abundance more than PSV6 and the mixed phage treatment (Figure 10A). Hence, leaf disease observations indicated that at an MOI of 100, the severity of leaf symptoms followed the order of PSV6 > mixed treatment > PSV3. Moreover, the leaf disease symptoms in each group at an MOI of 100 were less severe than those observed at MOI 1 and MOI 10 and those in the negative control group (Figure 10B).

## 4 Discussion

The rapid emergence of antibiotic-resistant bacteria, coupled with the slow pace of new antibiotic development, has greatly intensified the search for alternative methods. As a result, there is a growing demand for sustainable and environmentally friendly alternatives to chemical pesticides (Holtappels et al., 2021). Therefore, in the present study, we isolated and characterized two novel phages from wastewater samples, PSV6 and PSV3, and explored their potential as biocontrol agents for the management of *P. syringae* infections. These phages exhibited strong lytic activity against Pss, a notorious pathogen that causes bacterial disease in plants. Our findings indicated that they may be safe and effective alternatives for treating bacterial infections in plants.

Sewage is a rich reservoir of phages, from which many phages have been isolated. Notable examples include *Vibrio alginolyticus* phage vB_ValA_R15Z (Li et al., 2024), *Aeromonas hydrophila* phage vB-AhyM-AP1 (Pallavi et al., 2021), and *Acinetobacter baumannii* phage ϕkm18p (Shen et al., 2012). Similarly, PSV6 and PSV3 were also isolated from sewage in the present study. These phages formed clear, distinct plaques on Pss double-layer agar plates. PSV6 (1–3 mm) produced large, clear middle plaques, similar in size to those formed by *Pseudomonas* phage vB_PsyM-PHB09 (approximately 2 mm), which was isolated from kiwifruit in Sichuan (Liu et al., 2021). In contrast, PSV3 (0.5–1 mm) produced comparatively smaller plaques than those of PSV6. However, both phages formed plaques smaller than those of the 16 *Pseudomonas syringae pv. phaseolicola* phages (approximately 5 mm) isolated from soil (Martino et al., 2021). Our TEM analysis revealed that both phages belong to the order *Caudoviricetes*, with PSV6 exhibiting a short-tailed podovirus-like morphology and PSV3 displaying a long-tailed siphovirus morphology. This morphological distinction further highlights the diversity of phages that can infect Pss and provides insights into the structural features that may influence the efficiency of host recognition and infection. Moreover, the distinct tail morphologies of these two phages essential for their use as biological control agents. The MOI is an inherent property of phages; hence, its effectiveness in microbial inactivation is critical. In the present study, the optimal MOI of PSV6 and PSV3 was 1. In comparison, the optimal MOIs of the Pss phage SoKa (Oueslati et al., 2022) and phage ϕ6 (Pinheiro et al., 2020) are 0.01 and 0.001, respectively. In terms of the growth characteristics of the two phages isolated in the present study, PSV6 (135 PFU/cell) exhibits a larger burst size than that of PSV3 (16 PFU/cell). This indicates that PSV6 has a higher replication efficiency in Pss than does PSV3. Moreover, the burst size of PSV6 is significantly larger than that of the ϕ6 phage (60 ± 1 PFU/cell), which is also a *P. syringae* pv. *syringae* phage (Pinheiro et al., 2019). In contrast, the burst size of PSV3 is smaller than that of the ϕ6 phage. Moreover, PSV3 required a longer latent period (45 min) than PSV6, which is reflected in the lysis curve as a delayed reduction in the Pss OD value compared to PSV6.

Another critical challenge in the development of phage-based biocontrol agents is their stability under different environmental conditions (Sae-Ueng et al., 2024), particularly different pH levels (Zhang et al., 2015), temperatures (Jończyk et al., 2011), and solvents (Nakayinga et al., 2021). These conditions can significantly affect the adsorption, infectivity, intracellular replication, and proliferation of phages. Our results revealed that both PSV6 and PSV3 exhibited a broad range of environmental tolerances, which is essential for their practical application in agriculture. Notably, some phages can only proliferate under narrower condition ranges. For example, the optimal conditions of phage vB_EliS-R6L are a pH range of 7–10 and temperatures below 40° C (Lu et al., 2017). However, PSV3 exhibited greater stability under extreme conditions than PSV6 and Psa phage ϕPSA2 (pH 5–9, temperature ≤ 50° C) (Fiorillo et al., 2023). These results indicate that PSV6 and PSV3 can be used in fields where the pH values of the soil and irrigation water fluctuate. The most critical period for plant growth is from autumn through winter to early spring, when temperatures are lower (Liu et al., 2021). Therefore, temperature is not a limiting factor for the implementation of these two phages in agricultural applications, as they are likely to maintain their infectivity under typical environmental conditions. We also assessed the susceptibilities of these phages to organic solvents. Although both PSV6 and PSV3 exhibited some degree of sensitivity to solvents such as chloroform, acetone, and ethanol, they remained viable under certain conditions. This finding suggests that formulations of these phages could be developed to enhance their resistance to environmental stressors. These formulations could help prolong the shelf life of these phages and improve their efficacy in field applications. Moreover, the host specificity of bacteriophages is a core characteristic that enables their unique role in nature and human applications. Phages PSV6 and PSV3 exhibit strong host specificity, allowing them to specifically target their host bacteria in natural environments without directly affecting the density, diversity, or vitality of other microorganisms within the microbial community.

Genome sequencing and phylogenetic analyses revealed that PSV6 and PSV occupy distinct branches, confirming them as novel phages. Furthermore, the two phages are distantly related. The fact that phages isolated from the same host are distantly related may be attributed to the high mutability and recombination potential of the host recognition module, which encodes the phage injection mechanism and receptor-binding proteins (Gonzalez-Serrano et al., 2023). This adaptability helps maintain their infectivity toward co-evolving bacterial hosts. Additionally, despite significant differences in the core genes of the two phages, they may still acquire the ability to infect the same host through horizontal gene transfer. Moreover, phages are potentially important natural reservoirs for the transmission of antibiotic resistance and virulence genes (Modi et al., 2013; Calero-Cáceres and Muniesa, 2016). However, our genome analysis revealed that PSV6 and PSV3 lack known virulence factors and antibiotic resistance genes. This suggests that these phages do not harbor genes that could pose risks to the environment, human health, or agricultural systems, making them suitable candidates for biocontrol applications. Furthermore, the lack of antibiotic resistance genes mitigates concerns regarding the spread of resistance in pathogenic bacteria, which is a growing issue with the widespread use of antibiotics in agriculture.

Although phages represent a promising alternative to antibiotics, the development of phage resistance remains a major challenge (Hatfull et al., 2022). Our results showed that the higher the titers of PSV6 and PSV3, the earlier resistance emerged. The occurrence of resistance at higher MOIs may be related to stronger selective pressure, which could accelerate the emergence of resistant bacterial strains. The emergence of bacterial resistance is often addressed through phage cocktail therapy (Topka-Bielecka et al., 2021; Mirzaei et al., 2022). However, in some cases, certain phages within a cocktail may compete with one another, reducing their overall effectiveness compared to single-phage treatments. Our experiments demonstrated that, compared with PSV3 alone, the use of phage mixtures at an MOI of 100 for 15 min resulted in reduced effectiveness, consistent with previous findings (Schmerer et al., 2014). This phenomenon is likely attributable to differences in the infection mechanisms of individual phages or to unfavorable interactions during treatment, which may impair their activity. Furthermore, our results revealed contrasting trends in bactericidal efficiency between *in vitro* and *in vivo* experiments. *In vitro*, treatment with phages at an MOI of 1 achieved the highest bactericidal efficiency and delayed the onset of resistance. In contrast, *in vivo*, increasing the phage concentration significantly reduced bacterial numbers. This suggests that higher phage concentrations increase the likelihood of contact with a larger number of bacterial cells, thereby enhancing the treatment effect. These findings underscore the importance of carefully selecting and optimizing phage combinations to minimize interference between phages and maximize their collective therapeutic effect. Additionally, optimizing phage dosage, delivery methods, and treatment timing should be key priorities for future research aimed at improving the efficacy of phage therapy.

Although our study using *A. thaliana* as a model system provides valuable experimental data for phage-based biocontrol research, it is important to acknowledge that agricultural crops differ substantially from *Arabidopsis* in terms of morphology, physiology, and immune mechanisms. Therefore, validating phage treatments on relevant agricultural host plants is essential. Moreover, the experimental methods and cultivation conditions in this study were conducted under controlled laboratory settings, which do not fully replicate the complex microecological interactions between microbes and plants in natural environments. Under field conditions, factors such as soil type, humidity, and plant variety can influence phage stability, dispersion, and interactions with plant pathogens, ultimately affecting treatment outcomes. Thus, further research is required to evaluate the performance of these phages in field trials.

While phage therapy has been successfully applied in various fields, including medicine, agriculture, food production, environmental management, and aquaculture (Liu et al., 2022b), its development and application in plant disease management still face several challenges (Jończyk-Matysiak et al., 2019). Nevertheless, our findings provide strong support for the potential use of phages in real agricultural environments and offer valuable insights for future research. We believe that phages could become a vital tool for plant disease management in agriculture moving forward.

## 5 Conclusion

In conclusion, phages PSV6 and PSV3 are two novel Pss-specific phages with strong potential for phage-based control of *P. syringae*. Future studies should focus on optimizing formulation strategies and conducting field trials to validate their efficacy, thereby advancing phage therapy as a means to reduce reliance on chemical pesticides and promote sustainable agricultural practices.

## Data Availability

The datasets presented in this study can be found in online repositories. The genome sequences of phages PSV6 and PSV3 have been deposited in GenBank (https://www.ncbi.nlm.nih.gov/genbank), under the accession numbers OP485124 and OP712460, respectively.
