# Supplementary figures and images for: Characterization of two novel bacteriophages (PSV6 and PSV3) as biocontrol agents against Pseudomonas syringae
Source: Front Microbiol. 2025 Nov 12;16:1633072. doi: 10.3389/fmicb.2025.1633072 (PMC12651447; doi:10.3389/fmicb.2025.1633072)

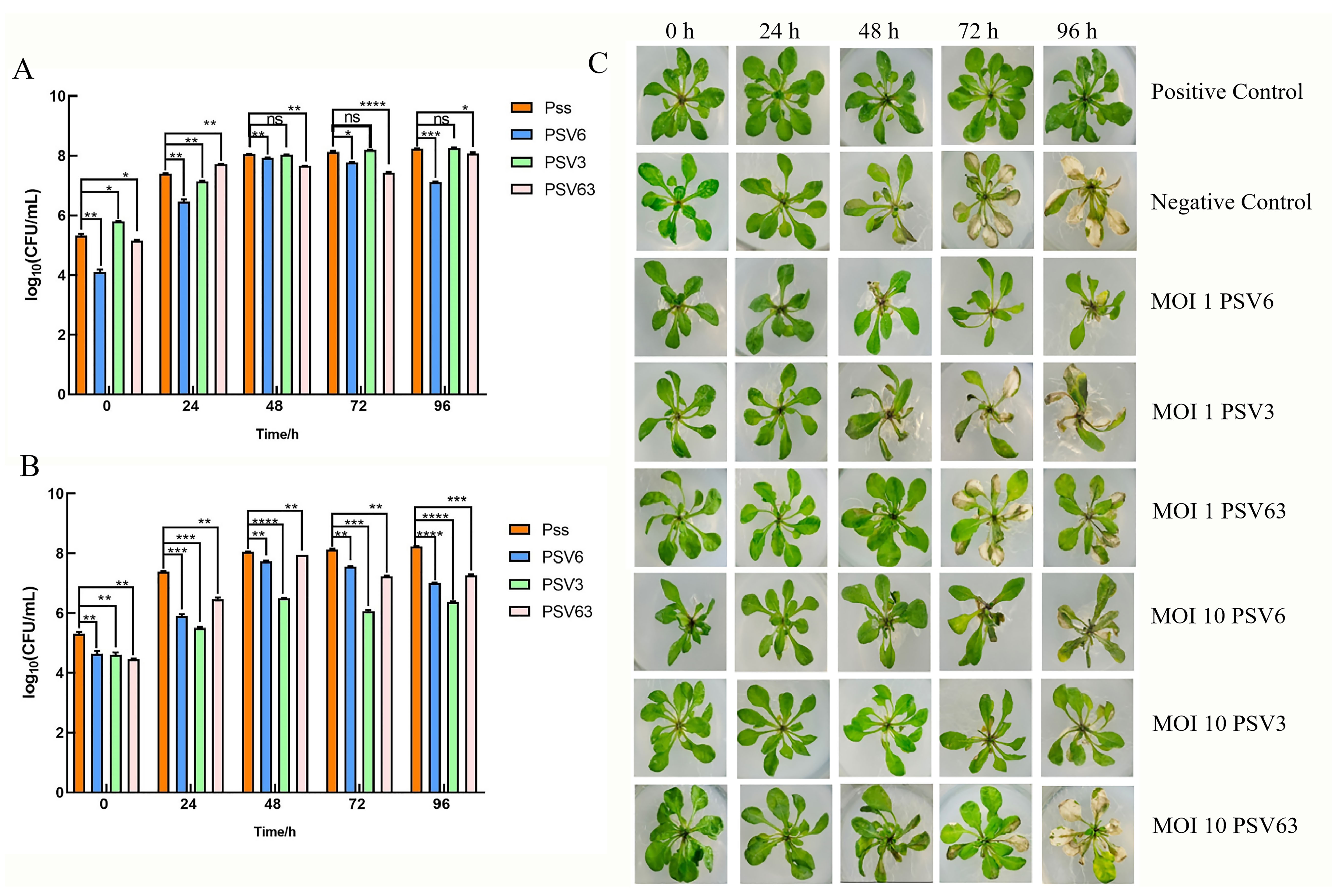

Supplement: Supplementary file 1 [file Image_1.jpeg]
